# Co-delivery of Bmi1 small interfering RNA with ursolic acid by folate receptor-targeted cationic liposomes enhances anti-tumor activity of ursolic acid *in vitro* and *in vivo*

**DOI:** 10.1080/10717544.2019.1645244

**Published:** 2019-08-01

**Authors:** Weijie Li, Ruicong Yan, Yong Liu, Chuanchuan He, Xiaojuan Zhang, Yao Lu, Muhammad Waseem Khan, Chuanrui Xu, Tan Yang, Guangya Xiang

**Affiliations:** a Department of Pharmacy, Tongji Hospital, Tongji Medical College, Huazhong University of Science and Technology, Wuhan, P. R. China;; b School of Pharmacy, Tongji Medical College, Huazhong University of Science and Technology, Wuhan, P. R. China

**Keywords:** Liposome, Bmi1 siRNA, ursolic acid, folate receptor, tumor targeting

## Abstract

Overexpression of Bmi1 gene is an important feature of cancer stem cell in various human tumors. Therefore, Bmi1 gene can be a potential target for small interfering RNA (siRNA) mediated cancer therapy. Ursolic acid (UA) as a natural product plays a pivotal role in anti-tumor field, although its performance is limited by low bioavailability and poor hydrophilicity. A folate receptor-targeted cationic liposome system was designed for the purpose of investigating the relationship between Bmil siRNA and UA. The folate receptor-targeted cationic liposomes co-delivering UA and Bmi1 siRNA (FA-UA/siRNA-L) were fabricated by electrostatic interaction between folate UA liposome (FA-UA-L) and Bmi1 siRNA. Tumor growth is inhibited by FA-UA/siRNA-L *in vitro* and *in vivo* and this inhibition is contributed by a synergistic anti-tumor effect of UA and Bmi1 siRNA. The western blot measurement of apoptosis-protein and cancer stem cell (CSC) marked-protein demonstrated that UA led to activation-induced tumor cell death and Bmi1 siRNA resulted in inhibition of cancer stem cells. Overall, these results indicate that Bmi1 as a regulating gene for cancer stem cell is an effective target for cancer treatment using siRNA and co-delivery of UA and Bmi1 siRNA using folate-targeted liposomes is a promising strategy for improved anti-tumor effect.

## Introduction

It is widely acknowledged that small interfering RNA (siRNA) plays a key role in gene therapy and can be potentially applied in clinical practice. Recently, the use of siRNA to down-regulate the expression of oncogenes has become an attractive strategy to inhibit the proliferation of tumor (Oh and Park, 2009; Zhou et al., 2018). Bmi1 as a member of polycomb repressive complex 1 (PRC1) provides important information on self-renewal and malignancy of stem cell (Gray et al., 2016). Up-regulation of Bmi1 gene has been explored in several tumor types, such as nasopharyngeal carcinoma, non-small cell lung carcinoma, medulloblastoma, colon carcinoma, and metastatic melanoma (Zacharek et al., 2011; Wang et al., 2013; Ferretti et al., 2016; Zhang et al., 2016; Bakhshinyan et al., 2018). Several recent studies on cancer stem cells have shown that down-regulation of Bmi1 could inhibit the tumor cell growth in different types of cancer, indicating that Bmi1 can be a potential target for tumor treatment (Liang et al., 2013; Zhu et al., 2014). Although the molecular mechanism of using siRNA to down-regulate Bmi1 expression has been reported (Gargiulo et al., 2013), the biological applicability and clinical translation of Bmi1 siRNA based therapy are still limited. This is mainly because siRNA showed poor internalization into tumor cells, fast systemic clearance and was susceptible to enzymatic degradation (Hahn et al., 2004; Hoerter et al., 2011).

Over the past decade, it has been revealed that cancer could not be effectively treated through monotherapy since cancer is a result from interconnected diseases with combination of pathways (Woodcock et al., 2011). Single chemotherapy often leads to cancer relapse and drug resistance on account of anti-target activities, compensatory actions, neutralizing actions, cross-talk, and pathway redundancy (Jia et al., 2009). Combining siRNA with chemotherapeutic drug has been proved to be feasible for enhanced anti-tumor activity (Morris, 2003; Prados et al., 2012). However, due to the instability of siRNA and poor solubility related to drugs, a proper co-delivery system is needed. Previous studies have shown the potential of using nanocarriers to co-deliver chemotherapeutic drugs and siRNA (Chen et al., 2012; Nishida et al., 2016; Zhou et al., 2018). Minko *et al*. have shown that liposomal co-delivery of siRNA and doxorubicin (DOX) targeting multidrug resistant (MDR) cancer cells enhanced the anti-tumor efficacy of DOX (Saad et al., 2008). Moreover, micelleplex nanoparticles have been reported as co-delivery systems for siRNA and paclitaxel (PTX) to promote synergistic tumor suppression (Sun et al., 2011). Our previous studies have also reported two co-delivery systems combining Bmi1 siRNA with DOX or cisplatin (CPT) (Yang et al., 2014; Yang et al., 2018). In general, most of nanocarrier-mediated co-delivery systems reported were designed to enhance the anti-tumor effect of first-line chemotherapy drugs such as DOX, PTX, CPT, and others. However, the co-delivery of siRNA and natural compounds with anti-tumor effects by drug delivery systems has rarely reported.

Ursolic acid (UA; 3β-hydroxy-12-urs-12-ene-28-oic acid), a pentacyclic triterpenoid carboxylic acid, can be derived from many plants and constitutes the main components of some Chinese herbal medicine. Recently, there have been increasing interests in the biological functions of UA, such as anti-inflammation, anti-oxidative and anti-tumor activities (Hussain et al., 2017,Chen et al., 2018). One of the most important functions of UA is the anti-tumor effects on many types of cancers through activating apoptosis pathway and thereby inducing tumor cell death. Xiang et al. demonstrated that the early metastasis and recurrence of hepatocellular carcinoma (HCC) could be reduced by UA and its prodrug (Xiang et al., 2015). Tang et al. found that leading through influence the pathway of EMT and EGFR could reduce lung metastasis by aspirin and UA co-drug (Tang et al., 2016). Moreover, Dong et al. showed that glucose metabolism of tumor cells could be decreased by the combination of 2-deoxy-d-glucose and UA (Dong et al., 2015). Although the extreme safety of UA in cancer treatment has been testified in the clinical trial, the poor solubility of UA still limits its application as an anti-cancer agent (Zhu et al., 2013).

Despite the critical role of UA or Bmi1 siRNA in cancer treatment individually, the use of nanocarrier to co-deliver both of them as a combination therapy has not been reported. This paper describes the design and implantation of folate-targeted cationic liposomes co-delivering Bmi1 siRNA and UA (FA-UA/siRNA-L). The purpose of this study was to explore the relationship between UA and Bmi1 siRNA and to detect the property of folate-target cationic liposome. UA was encapsulated into liposomes and its solubility was improved, Bmi1 siRNA was absorbed on the surface of liposomes, which enhanced the stability of the siRNA. In addition, functionalizing the liposomes with folate-targeted ligands increased the active tumor-targeting effect. The performance of FA-UA/siRNA-L was evaluated *in vitro* by cellular uptake, cell growth inhibition, gene expression, and apoptosis analysis. Also, the anti-tumor efficacy of FA-UA/siRNA-L in the KB xenograft model of nude mice was assessed.

## Material and methods

### Material

UA and folate-PEG-CHEMS were obtained from the pharmacy school of Tongji Medical College, Huazhong University of Science and Technology, Wuhan, China (purity >95%). 1, 2-dioleoyl-3-trimethylammonium-propane (Chloride Salt) (DOTAP) was purchased from Avanti Polar Lipids Inc. (Alabaster, AL, USA). Monomethoxy polyethylene glycol 2000-distearoyl phosphatidylethanolamine (MPEG-DSPE2000) was obtained from Lipoid GmbH (Germany). Cholesterol (CHOL) was purchased from Acros Organics. Calcein was obtained from Aladdin Chemistry Co. Ltd. Folic acid was purchased from Sinopharm Chemical Reagent Co., Ltd. RPMI 1640 media were purchased from Sigma-Aldrich Corp. (St. Louis, MO, USA). Methyl thiazolyl-diphenyl-tetrazolium bromide (MTT). Fetal bovine serum (FBS) was purchased from Zhejiang Tianhang Biological Technology Co., Ltd., Hangzhou, China. Female Kunming mice (25 g, 6–8 weeks old) and female Balb/c nude mice (20 g, 6–8 weeks old) were obtained from Beijing Huafukang Bioscience Technology Co. (Beijing, China; license SCXK (jing) 2009-0004). Cy3-labeled Bmi1 siRNA were synthesized by Ribobio Co., Ltd (Guangzhou, China). The Bmi1 siRNA sequences for mice were sense strand 5'-CCA GAC CAC UCC UGA ACA UTT-3' and anti-sense strand 5'-AUG UUC AGG AGU GGU CUG GTT-3', and for human were sense strand 5'-CCA GAC CAC UAC UGA AUA UAA-3' and anti-sense strand 5'-UUA UAU UCA GUA GUG GUC UGG UU-3'.

### Cell culture

KB and LO2 cells were purchased from the China Center for Type Culture Collection of Wuhan University (Wuhan, China). The cells were cultured with high glucose or DMEM folate-free RPMI 1640 medium with streptomycin, penicillin, and 10% FBS solution in 37 °C and 5% CO_2_ incubators.

### Preparation of FA-UA/siRNA-L nano-liposome

The liposomes were prepared using thin-film dispersed hydration method according to the previous study (Xiang et al., 2008). The lipid composition of folate receptor-targeted UA liposome (FA-UA-L) was DOTAP/CHOL/MPEG-DSPE2000/FA-PEG-CHEMS at a molecular ratio of 40:55:4.5:0.5 (at weights of 28, 21.3, 12.6, and 2.1 mg). UA to lipid ratio is 1:20 (w/w). The lipid and UA was dissolved in chloroform and then dried at 40 °C using the rotary evaporator under reduced pressure to form the thin lipid film. The resulting lipid film was hydrated with PBS (pH 6.8) at 60 °C for 30 min to form preliminary liposomes. The large multilamellar liposomes were extruded through a polycarbonate membrane (0.2 μm pore size) for 12 times to obtain nano-sized and unilamellar liposomes. The positively-charged liposomes adsorbed negatively-charged Bmi1 siRNA by electrostatic interaction at a ratio of more than 200:1 (w/w, liposome/siRNA) in RNase free H_2_O to form FA-UA/siRNA-L.

### Encapsulation efficiency

After final solution of FA-UA/siRNA-L was prepared, the unencapsulated UA was removed through low-speed centrifugation (3000 *g*) at room temperature for 10 min. The liposomes collected in the supernatant were lysed in methanol and the released UA was determined by HPLC with a UV wavelength of 210 nm. The entrapment efficiency was calculated as the ratio of amount of liposome-encapsulated UA to amount of UA initially added (Liu et al., 2006).

### Characterization of FA-UA/siRNA-L nano-liposome

Physicochemical characteristics of liposomes were determined using a Zeta PALS instrument (DLS, Zeta Plus, Brookhaven Instruments, USA) conform to the specification. Each sample was detected 3 times at room temperature and the average values and standard deviations were calculated.

Atomic Force Microscope (AFM) was used to determine the shape of liposomes. Mica was used to disperse the liposome solution at a volume of 5 μL. After 10 min incubation, the surface of mica was rinsed with DI water 4 times and dried with nitrogen. Then, the condensated samples were determined by Atomic Force Microscope (Nanoscope III A, Digital Instruments, Veeco MultiMode).

### Uptake of FR-Targeted liposome assay

KB cells were seeded in 12-well plate with folate-free RPMI 1640 medium and cultured for 24 h. In order to block the folate receptor, 1 mM free folate was first added to the medium. After washing the KB cells grown in the plates with the medium for 3 times, Calcein/siRNA-L (Cy3 labeled) or FA- Calcein/siRNA-L (Cy3 labeled) was added and incubated with the cell for 1 h at 37 °C. Then, the KB cells were washed by PBS 5 times and 4% paraformaldehyde solution was used to fix the cells for 15 min. The cell nucleus was stained with 4,6-Diamidino-2-phenylindole (DAPI) solution and the uptake of FR-targeted liposomes was determined by a fluorescence microscope (Olympus, Japan).

### Real-time PCR

To determine the gene silencing efficiency, the cells were transfected respectively with FA-UA/siRNA-L and UA/siRNA-L. The KB cells were collected after incubating for 48 h and washed with PBS 5 times. Then, total RNA extraction was applied with Trizol reagent. Reverse Transcription System (Promega, Madison, USA) was used to synthesize cDNA. The CFX96 Real-Time PCR detection system with SYBR Green was used to measure Bmi1 mRNA.

### Western blot analysis

The KB cells (8 × 10^5^ per well) were seeded in 4-well plate and treated with related liposomes and then incubated in CelLytic M Cell Lysis Reagent (Sigma-Aldrich, MA, USA) for 30 min on ice. BCA assay was used to quantify and extract protein. The supernatant was collected after centrifugation at 12,000 rpm (Eppendorf 5415 D). Cell lysates were separated on a 10% polyacrylamide gel and transferred to a PVDF membrane. The membrane was blocked for 1 h in 5% skim milk and then incubated with monoclonal antibody against Bmi1 (1:1000, Millipore, MA, USA), CD133 (1:1000, Abcam, Cambridge, UK) , Caspase 9 (1:1000, Cell Signaling), or tubulin (1:1000, Abcam) overnight. The membrane was then washed in TBST (PBS with 0.1% Tween-20) 3 times and then incubated for 1 h with secondary antibody. Then the membrane was washed 4 times and developed by an enhanced chemiluminescence system according to the manufacturer’s instructions (Perkin Elmer, Waltham, MA, USA).

### Cytotoxicity

The cytotoxic effect of related formulations against the tumor cells was evaluated by MTT assay. KB cells were first seeded in 96-well plates at a density of 8 × 10^3^ cells per well and cultured for 24 h, after which FA-UA/siRNA-L, FA-siRNA-L, FA-UA-L, UA-L, or free UA (with the final Bmi1 siRNA concentration of 100 nM and UA concentration of 50 uM) was added in the culture medium. After another 24 h incubation, the proportion of living cells were measured by the colorimetrical method according to the user’s manual.

### In serum stability of FA-UA/siRNA-L

To determine whether FA-UA/siRNA-L is stable in blood circulation, FA-UA/siRNA-L was incubated with serum (1 h, 3 h, and 6 h) and transfected to KB cells for 1 h. Then, the KB cells were washed by PBS 5 times after the incubation and 4% paraformaldehyde solution was used to fix the cells for 15 min. The cell nucleus was stained with 4,6-Diamidino-2-phenylindole (DAPI) solution and detected by a fluorescence microscope.

### *In vivo* anti-tumor efficacy study

Subcutaneous KB tumor-bearing balb/c nude mice (6–8 weeks of age, 16–18 g) that obtained from Huafukang technology corporation (Beijing, China) was used to evaluate the anti-tumor effect of FA-UA/siRNA-L. When the tumor size reaches to 50–100 mm^3^ (the mice were fed with folate-free diet after the tumor implanted), saline, free UA, UA-L, FA-siRNA-L, FA-UA-L or FA-UA/siRNA-L was given intravenously through a tail vein five times every other day. The injection dose of each formulation was 4.5 mg/kg for UA and 170 μg/kg for siRNA. During the administration, the tumor volume and body weight of nude mice were measured regularly every 2 days. Tumor tissue and blood were collected after mice were sacrificed. Blood was used to detect serological markers (ALT, AST, and BUN) and tumor tissue was homogenized for H&E assay and western blot assay.

### Anchorage-independent growth assay

Cells were suspended in soft agar and growth medium in 6-well plates at a density of 5000 cells per well. After 2–3 weeks, colonies (6 cells per treatment) were counted under the microscope in 5 fields per well and photographed.

### Statistical analysis

GraphPad Prism 6 software was used to perform the statistical analysis. Multiple groups are compared by One-way ANOVA with Dunnett’s post-test. Comparison between groups was performed by Student’s *t*-test and one-way ANOVA with Dunnett’s *post-test*. Statistical significant difference was defined as *p < .05, **p < .01 and ***p < .001.

## Results

### Preparation and characterization of FA-UA/siRNA-L

The schematic diagram of FA-UA/siRNA-L (Figure S1A), the chemical structure of folate ligands (Figure S1B), siRNA sequence of Bmi1 (Figure S1C) and the chemical structure of UA (Figure S1D) were shown in Figure S1. The folate ligand structure was determined by mass spectrum. The centered distribution curve was at 4166 Da, consistent with the calculated molecular weight of 4158 Da (Figure S2). These results revealed that the folate ligand was successfully added onto the liposomes.

**Figure 1.**
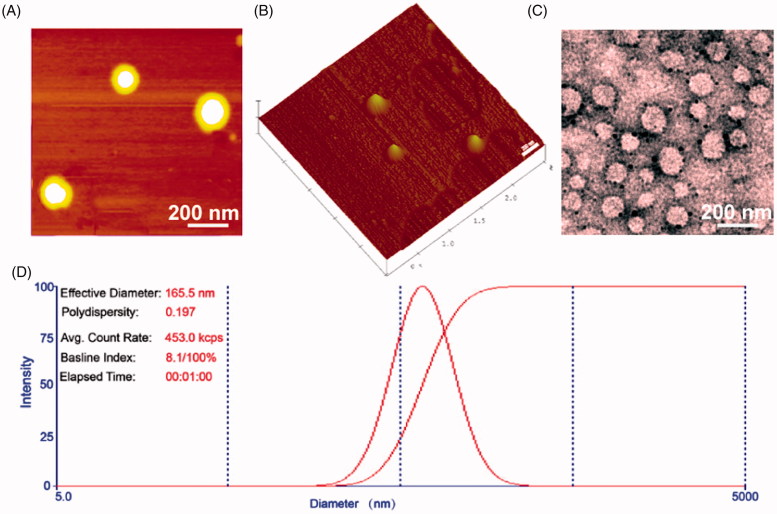
Characterization of FA-UA/siRNA-L. (A) and (B) Atomic Force Microscope image of FA-UA/siRNA-L. (C) The TEM image of the FA-UA/siRNA-L negative stained with phosphotungstic acid. (D) Size distribution of FA-UA/siRNA-L.

The average particle sizes of UA-L and F-UA-L were 134.5 and 139.1 nm, respectively. After Bmi1 siRNA was absorbed on the surface of liposomes, the average particle sizes were increased to 162.7 for UA/siRNA-L and 165.1 nm for FA-UA/siRNA-L. The zeta potential of UA-L and F-UA-L were 48.9 mV and 51.2 mV, respectively. The absorption of Bmi1 siRNA reduced the zeta potential of UA/siRNA-L and FA-UA/siRNA-L to 21.3 and 18.6 mV respectively. This is owing to that the positive charge of liposome was partly neutralized by negative charge Bmi1 siRNA. The entrapment efficiencies of UA in UA-L, F-UA-L, UA/siRNA-L, and FA-UA/siRNA-L were 79.8, 82.3, 77.9 and 81.9%, respectively (Table S1). The image of AFM revealed that the shape and size of liposomes were uniform (Figure 1(A,B)). Similar particle sizes (160 nm) were observed between TEM examination (Figure 1(C)) and DLS determination (Figure 1(D)). These results suggested that FA-UA/siRNA-L was prepared with good quality and could be used for the following *in vitro* and *in vivo* evaluations.

### Folate ligands increased targeted effect *in vitro*

**Figure 2.**
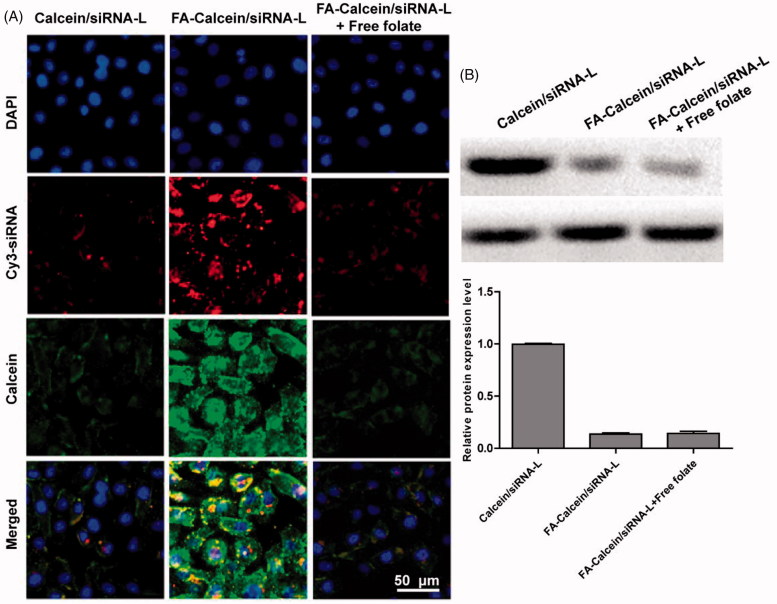
(A) In cellular uptake of Bmi1 siRNA and Calcein by FA-Calcein/siRNA-L in the KB cells. Bmi1 siRNA was labeled with Cy3 (red) and Calcein emits green fluorescence by itself. The nucleus was counterstained with DAPI (blue). (B) Western blotting assay of folate receptor protein expression in KB cells that treated with different formulations.

To investigate the active targeting effect of folate ligands with FA-UA/siRNA-L, Calcein that excited green fluorescence replaced the role of UA to determine the uptake effect. Calcein was loaded into liposomes and its surface absorbed with Cy-3 labeled Bmi1 siRNA that the liposome contained two fluorescence marker to use as uptake tracer. KB cells were used to incubate with liposomes and evaluated by fluorescence microscopy. As shown in Figure 2(A), compared to Calcein/siRNA-L, FA-Calcein/siRNA-L resulted in an enhanced uptake of both the Calcein (green) and Bmi1 siRNA (red) into the KB cells. However, the pretreatment of 1 mM free folate before adding FA-Calcein/siRNA-L did not increase the cellular uptake of Calcein and Bmi1 siRNA relative to Calcein/siRNA-L (Figure 2(A)). These results demonstrated that FA-UA/siRNA-L could efficiently deliver both Calcein and Bmi1 siRNA into KB cells through folate-targeted ligand. We examined folate receptor expression on KB cell surface after folate-targeted liposomes and folate free administration by WB detection. The results showed that the expression of receptors on the KB surface was inhibited after the folate-targeted liposome group was administered, suggested that the folate ligand could competitively bind to the folate ligand (Figure 2(B)).

**Figure 3.**
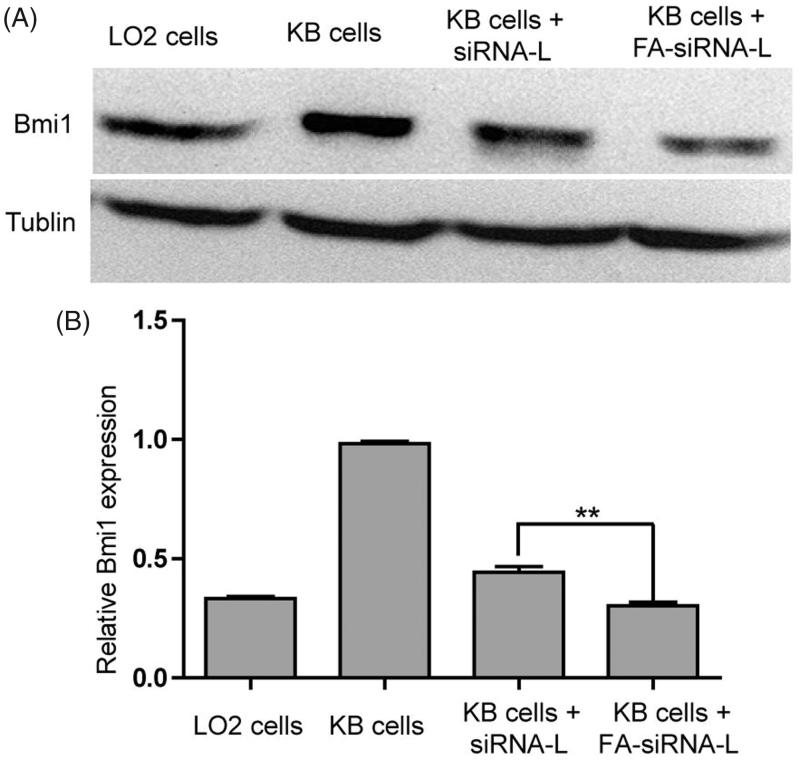
(A) Western blotting assay of Bmi1 expression in LO2, and KB cells that treated with different formulations. (B). RT-PCR assay of Bmi1 expression in LO2, and KB cells that treated with different formulations. Data are shown as mean ± SEM of 3 independent experiments. The statistical significant difference between two groups was defined as **p < .01.

Gene silencing effect of Bmi1 siRNA that delivered by siRNA-L and FA-siRNA-L were detected by western blotting and RT-PCR. To induce the influence of UA on Bmi1 gene, UA was removed from liposomes. As shown in Figure 3(A,B), the expression of Bmi1 gene in KB cells was higher compared with LO_2_ cells that labeled for normal cells. This phenomenon consisted of the report that Bmi1 gene was up-regulated in cancer cells and tissue (Sasaki et al., 2008; Xu et al., 2009; Biehs et al., 2013). After Bmi1 siRNA was delivered by siRNA-L or FA-siRNA-L, Bmi1 gene expression was greatly reduce. Interestingly, FA-siRNA-L showed stronger gene inhibition than siRNA-L (Figure 3(A,B)). These findings indicate that Bmi1 gene could be effectively down-regulated by Bmi1 siRNA that delivered by siRNA-L or FA-siRNA-L and liposomes that modified with folate ligands could intensify this effect.

### The improved cytotoxic effects of Bmi1 siRNA and UA delivered by FA-UA/siRNA-L

**Figure 4.**
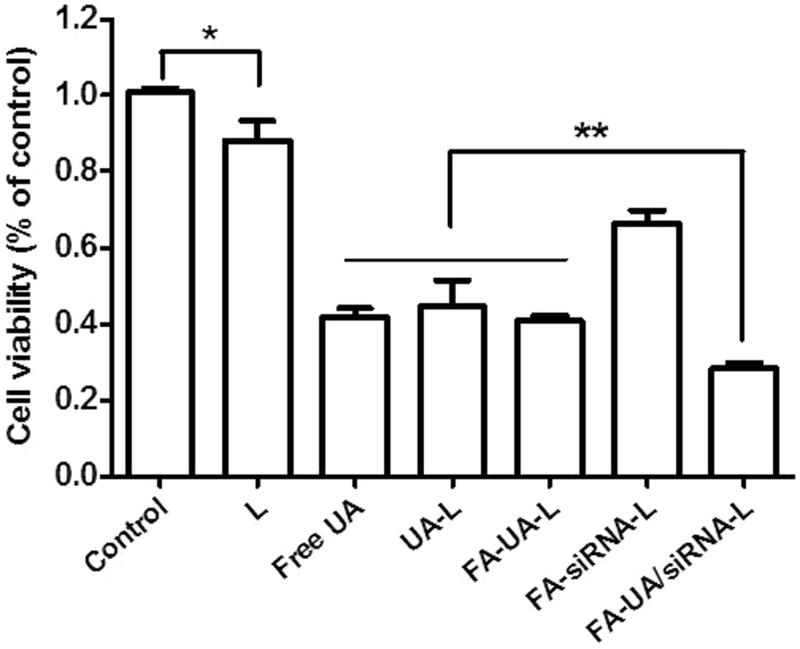
Cell viability of KB cells incubated with the different formulation with the final Bmi1 siRNA concentration of 100 nM and UA concentration of 50 uM. Data are shown as mean ± SEM of 3 independent experiments. The statistical significant difference between two groups was defined as. *p < .05; **p < .01.

In order to detect the synergistic anti-tumor effects of UA and Bmi1 siRNA delivered by FA-UA/siRNA-L, the cytotoxicity assay of various UA and Bmi1 formulations was measured in KB cells. As shown in Figure 4, there was a significant inhibition of cell growth found in FA-siRNA-L treated group compared to the control group (treated by saline). After incubating with FA- siRNA-L for 24 h, almost 30% of KB cells were dead. After incubated with UA-L for 24 h, 43% of KB cells were diminished. The free UA and FA-UA-L group showed similar cytotoxicity in KB cells with UA-L group. After treated with FA-UA/siRNA-L for 24 h, 71% inhibition of KB cells was observed. The results illustrated that in vitro study, there was a significant positive correlation between Bmi1 siRNA and UA co-delivered by folate-targeted liposomes to inhibit tumor cells and revealed enhanced cytotoxic effects.

### *In vivo* anti-tumor and toxicity study of FA-UA/siRNA-L

**Figure 5.**
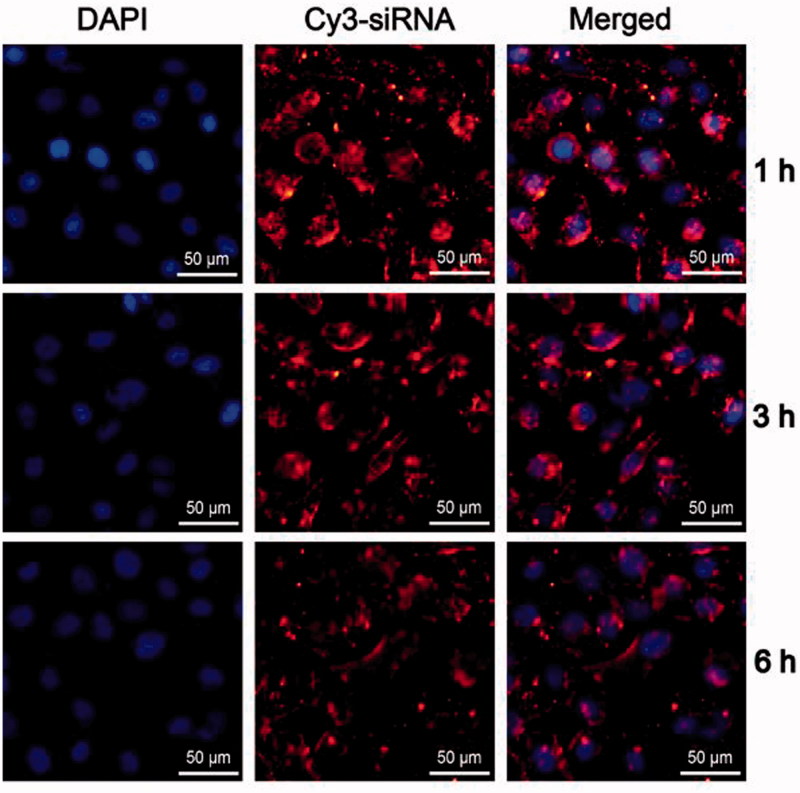
Stability of FA-UA/siRNA-L in serum. FA-UA/siRNA-L was incubated respectively with mice serum for 1, 3 and 6 h, and then examined the cellular uptake of the mixture to KB cells. The red fluorescence was emitted from Cy3 labeled Bmi1 siRNA. The nucleus was counterstained with DAPI (blue).

Before the evaluation of anti-tumor effect in vivo, we determined the stability of FA-UA/siRNA-L in serum in vitro. FA-UA/siRNA-L was incubated with serum (1 h, 3 h, and 6 h) and transfected to KB cells for 1 h. As shown in Figure 5, after 6 h culture with mouse serum, FA-UA/siRNA-L could still be uptaken by KB cells, reflected by the amount of Cy3 labeled Bmi1 siRNA not being reduced compared with that after 1 and 3 h culture with serum. Furthermore, Bmi1 siRNA delivered by folate-targeted liposomes also inhibited the expression of the Bmi1 gene after incubation with serum (Figure S3). This observation indicates that FA-UA/siRNA-L was stable in blood circulation and could be used further for *in vivo* anti-tumor study.

**Figure 6.**
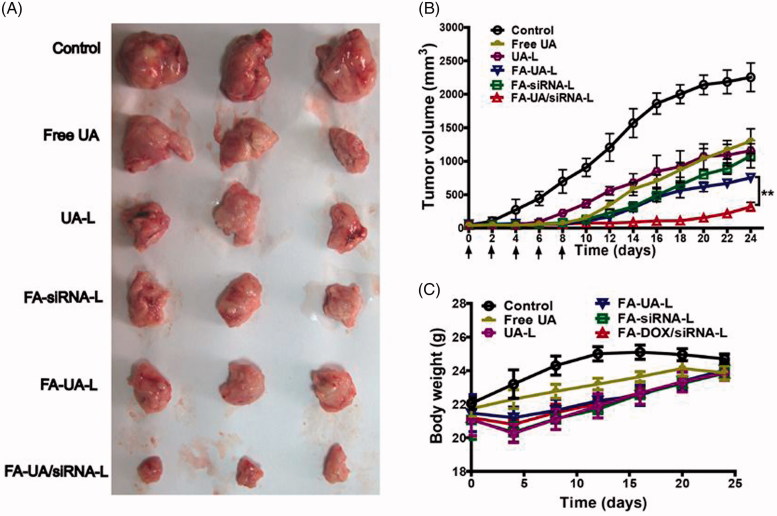
Anti-tumor efficacy of FA-UA/siRNA-L and the other formulations in the xenograft mouse model. (A). Tumor tissues obtained from mice that sacrificed on the 25^th^ day. (B). Growth curves of tumor volume determined during the treatment of saline, free UA, UA-L, FA-siRNA-L, FA-UA-L, and FA-UA/siRNA-L. Three per group of mice were respectively given i.v injection of different formulations by doses of 170 μg siRNA/kg, 4.5 mg UA/kg every other day for five times. The statistical significant difference between two groups was defined as. **p < .01. (C). Body weight changes curves of mice by the treatment with saline, free UA, UA-L, FA-siRNA-L, FA-UA-L and FA-UA/siRNA-L. Data are expressed as mean ± SD (*n* = 6).

To evaluate the anti-tumor effect of FA-UA/siRNA-L, human KB tumor xenograft nude mice were used. Various formulations loaded with UA and/or Bmi1 siRNA were divided into 6 groups (control, free UA, UA-L, FA-siRNA-L, FA-UA-L and FA-UA/siRNA-L). The mice of each group had freedom of movement and were in good spirits. They had a good appetite for food and water, and excretion was normal. After the growth of subcutaneous tumor attained to around 50–100 mm^3^, each formulation was administrated 5 times on every other day by tail vein injection. The mice were sacrificed after 25 days and tumor tissue was collected for detection of volume and biochemical. From the result, we could see that free UA group (1300 mm^3^) and UA-L group (1156 mm^3^) showed smaller tumors after the mice sacrificed at 25 days compared with the saline group (2254 mm^3^) (Figure 6(A)). Tumor growth curve consisted of this result (Figure 6(B)). Compared to UA and UA-L group, FA-UA-L group (753 mm^3^) and FA-siRNA-L group (1080 mm^3^) showed an even smaller tumor and stronger tumor growth inhibition (Figure 6(A,B)). The treatment of F-UA/siRNA-L group (318 mm^3^) demonstrated very significant tumor growth inhibition compared to other groups (Figure 6(A)). As the Figure 6(B) showed, there is a significant difference (*p* = .03) between F-UA/siRNA-L and FA-UA-L group. These results suggested that co-delivery of UA and Bmi1 siRNA by folate ligand modified liposomes could enhance the anti-tumor effect.

The body weight of mice was monitored to detect the systemic toxicity of FA-UA/siRNA-L. The mice were weighed every 4 days and the data was documented. As shown in Figure 6(C), the group of liposome injection revealed abrupt loss of body weight compared to UA and control group on the 4^th^ day. However, the weight resumed slowly after the injection of the 4^th^ day. For further safety evaluations of liposomes, biochemical index of AST, ALT (hepatic function index), BUN and CR (renal function index) were tested after injection of FA-UA/siRNA-L, FA-L, and saline. As shown in Figure S4A, both ALT and AST levels were significantly higher after injections of FA-L and FA-UA/siRNA-L injection compared to injection of saline. The AST/ALT ratios in the FA-L and FA-UA/siRNA-L groups were significantly lower compared to the saline group. These results suggest that the hepatic toxicity induced by liposomes was a mild and short-term effect. There were no significant differences in Blood Urea Nitrogen (BUN) and CR levels among the three groups, indicating low renal toxicity of liposomes (Figure S4B). Then, heart, kidney and liver tissues were taken out for H&E staining and no significant toxicity was found after injection of FA-UA/siRNA-L (Figure S5). All these findings show that FA-UA/siRNA-L had little and reversible systematic toxicity.

## Discussion

Many reports have elaborated the role of Bmi1 as an important regulator connected to self-renewal and maintenance of stem cells in many tissues (Alkema et al., 1997; Leung et al., 2004; Park et al., 2004). As a part of polycomb repressive complex 1 (PRC1), Bmi1 can target many genes through its function in modifying epigenetic chromatin (Bracken et al., 2007). Bmi1 also affected the occurrence and development of several types of cancer due to its influence on cancer stem cell-like phenotype (Bruggeman et al., 2007; Chiba et al., 2007). Tu et al. reported that according to overexpress miR-218 could reduce the glioma cell stem cell-like phenotype by targeting Bmi1. However, due to special functions of Bmi1 in maintaining hematopoietic stem cells and neuronal stem cells, the application and development of small molecular inhibitors targeted Bmi1 was limited by systemic toxicity. Indirect targeting RNA can be used to regulate Bmi1 gene expression, although an effective and safe delivery system is required to transport siRNA to tumor tissue. We reported in our previous study that folate-targeted cationic liposomes could efficiently transport Bmi1 siRNA to tumor tissue and resulted in enhanced anti-tumor effect when DOX was co-delivered (Yang et al., 2014).

In the current study, we continued to apply this mature folate-targeted system and for the first time examined the synergistic anti-tumor effect of Bmi1 siRNA with native compounds (UA). FA-UA/siRNA-L showed the best anti-tumor effect *in vitro* and *in vivo* compared to other formulations tested. To elaborate the mechanism of the synergistic anti-tumor effect of FA-UA/siRNA-L, we detected the expression of stem cell marker and apoptosis marker after the treatment of different formulations *in vitro* and *in vivo*. Bmi1 siRNA delivered by folate-targeted liposomes could down-regulate the expression of Bmi1 in KB cells and tumor tissues extracted from mice. Furthermore, the stem cell marker of CD133 expression was reduced by Bmi1 siRNA loaded liposome groups *in vivo* and *in vitro* as expected. Nevertheless, FA-siRNA-L and FA-UA/siRNA-L had little impact on the expression of apoptosis marker (Cleaved Caspase-9) *in vivo* and *in vitro* (Figure S6A and 5(B)). Anchorage-independent colony formation is a widely used method to evaluate Superior tumorigenesis or tumor initiating capability of CSC. Compared with non-siRNA treatment groups, siRNA treatment groups formed less colonies in soft agar, suggesting that Bmi1siR treatment significantly inhibited the colony formation ability of KB cells, indicating that the proliferation of CSCs were repressed (Figure S6C).

Although many reports suggested that the down-regulation of Bmi1 could block cell cycle and might cause apoptosis, these effects were far less impactful than tumor self-renewal (Chiba et al., 2007). Also, UA as an enhancement factor might also have induced tumor cell apoptosis, making the potential apoptosis promotion by Bmi1 siRNA less evident. As a tumor cell self-renewal inhibitor, Bmi1 siRNA could prevent the tumor relapse by inhibiting cancer stem cells. They could play anti-tumor effect role in the different pathway (Figure S7). Most studies of co-delivery systems have been designed for the purpose of enhancing the anti-tumor effect of first-line clinic chemotherapy drugs. The application of native compounds with potential anti-tumor effects has been rarely reported for combination with gene therapy. This study successfully combined gene therapy with native compounds and achieved the therapeutic goal.

As the most widely used nanodelivery systems, liposomes were used to deliver many kinds of drugs and have been approved in clinic therapy as commercial medicines (Sanhai et al., 2008). Liposomal encapsulation offers many advantages over unformulated chemotherapeutic drugs, such as enhanced tumor localization, overcoming multidrug resistance, prolonged systemic circulation and reduced side effect (Immordino et al., 2006). In recent decades, active targeting liposomes have been increasingly attractive for drug delivery. Compared to passive targeting liposomes, by adding ligands specifically binding to receptors overly expressed in the surface of cancer cells, active targeting liposomes could enhance cellular uptake and drug accumulation in tumors. As a GPI-anchored membrane protein, folate receptor was up-regulated in several kinds of tumor types (Ross et al., 1994). However, the expression of folate receptor in normal tissue cell was much lower than that in tumor cell. This phenomenon allows folate-receptor to be a potential target for nanodelivery systems. Therefore, many folate-targeted ligands were used to functionalized liposomes and any other nanocarrier drug delivery systems (Xiang et al., 2008; Jiang et al., 2009). In our previous and this study, we also successfully designed folate-targeted ligands as active targeting moieties (Huang et al., 2014). The cellular uptake of Bmi1 siRNA could be substantially enhanced through Folate-targeted liposomes delivery (Figure 2), suggesting the effectiveness of folate-targeted ligands.

## Conclusion

In this study, folate-targeted cationic liposomes co-delivering UA and Bmi1 siRNA (FA-UA/siRNA-L) were developed and evaluated *in vitro* and *in vivo*. UA and Bmi1 siRNA delivered by folate-targeted liposomes could be efficiently uptake by tumor cells *in vitro*. Furthermore, FA-UA/siRNA-L showed synergistic antineoplastic effect of *in vitro* and *in vivo*. Finally, we discussed the mechanism that Bmi1 as the cancer stem cell self-renewal target could be applied to the combination therapy with natural compounds. A further study with the focus on combination therapy of Bmi1 gene target is, therefore, suggested.

## Supplementary Material

Supplemental Material
