# Supplementary material for: Co-delivery of Bmi1 small interfering RNA with ursolic acid by folate receptor-targeted cationic liposomes enhances anti-tumor activity of ursolic acid in vitro and in vivo
Source: Drug Deliv. 2019 Aug 1;26(1):794–802. doi: 10.1080/10717544.2019.1645244 (PMC6711155; doi:10.1080/10717544.2019.1645244)
Supplement: Supplemental Material [file IDRD_A_1645244_SM6712.docx]

**Supplementary data:**

**Supplement table 1. Physicochemical properties of UA-L FA-UA-L UA/siRNA-L and FA-UA/siRNA-L**

| Liposome | Diameter (nm) | Zeta (mV) | PDI | EE (%) DL (%) |
| --- | --- | --- | --- | --- |
| UA-L | 134.5 ± 14.1 | 48.9 ± 4.2 | 0.194 ± 0.069 | 79.8 ± 11.2 4.3 ± 0.5 |
| FA-UA-L | 139.1 ± 12.5 | 51.2 ± 8.4 | 0.163 ± 0.065 | 82.3 ± 14.8 3.9 ± 0.4 |
| UA/siRNA-L | 162.7 ± 16.7 | 21.3 ± 6.1 | 0.184 ± 0.082 | 77.9 ± 13.4 3.6 ± 0.4 |
| FA-UA/siRNA-L | 165.1 ± 13.8 | 18.6 ± 4.6 | 0.197 ± 0.065 | 81.9 ± 17.5 3.4 ± 0.4 |

Note: each value represents the mean ± SD (n=3).

Abbreviations: EE, encapsulation efficiency of UA; PDI, polydispersity index; DL, Drug loading


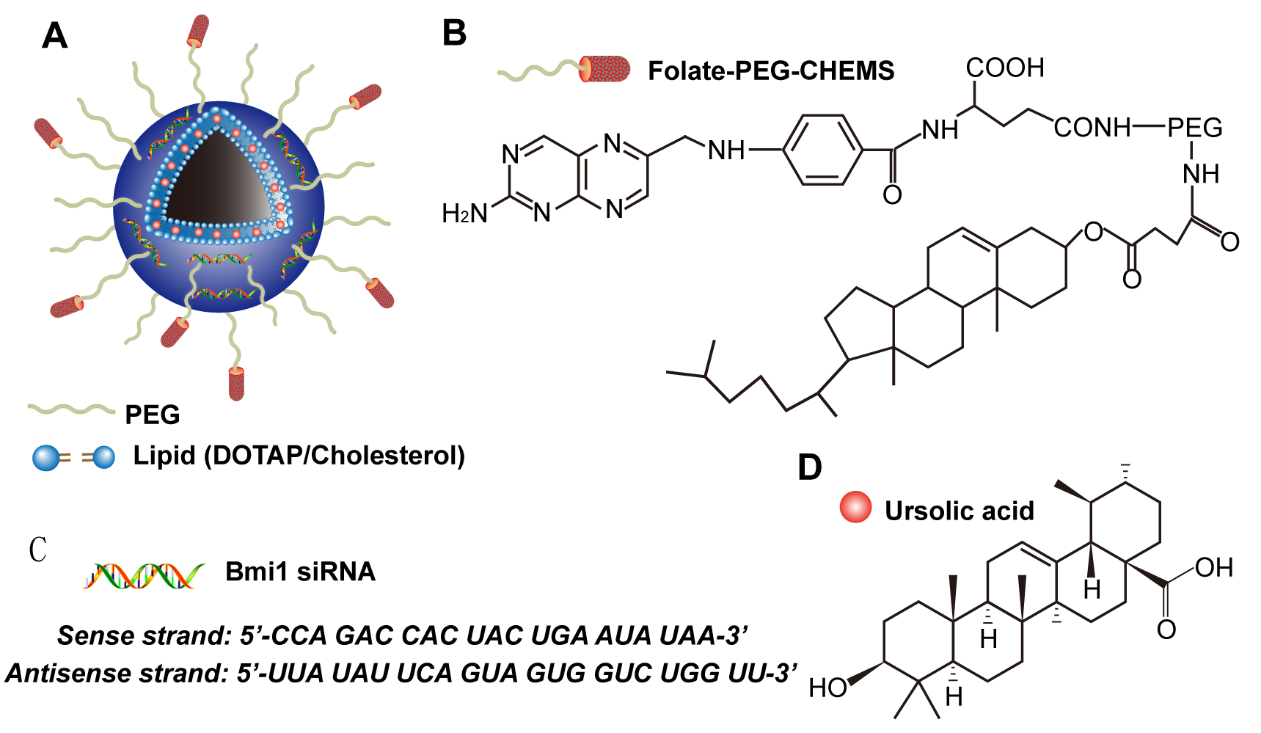


**Supplement figure 1.** Structure and composition of FA-UA/siRNA-L. (A) The schematic of FA-UA/siRNA-L structure. (B) The molecular formula of folate-targeted ligands. (C) The sequence of Bmi1 siRNA. (D) The molecular formula of UA.


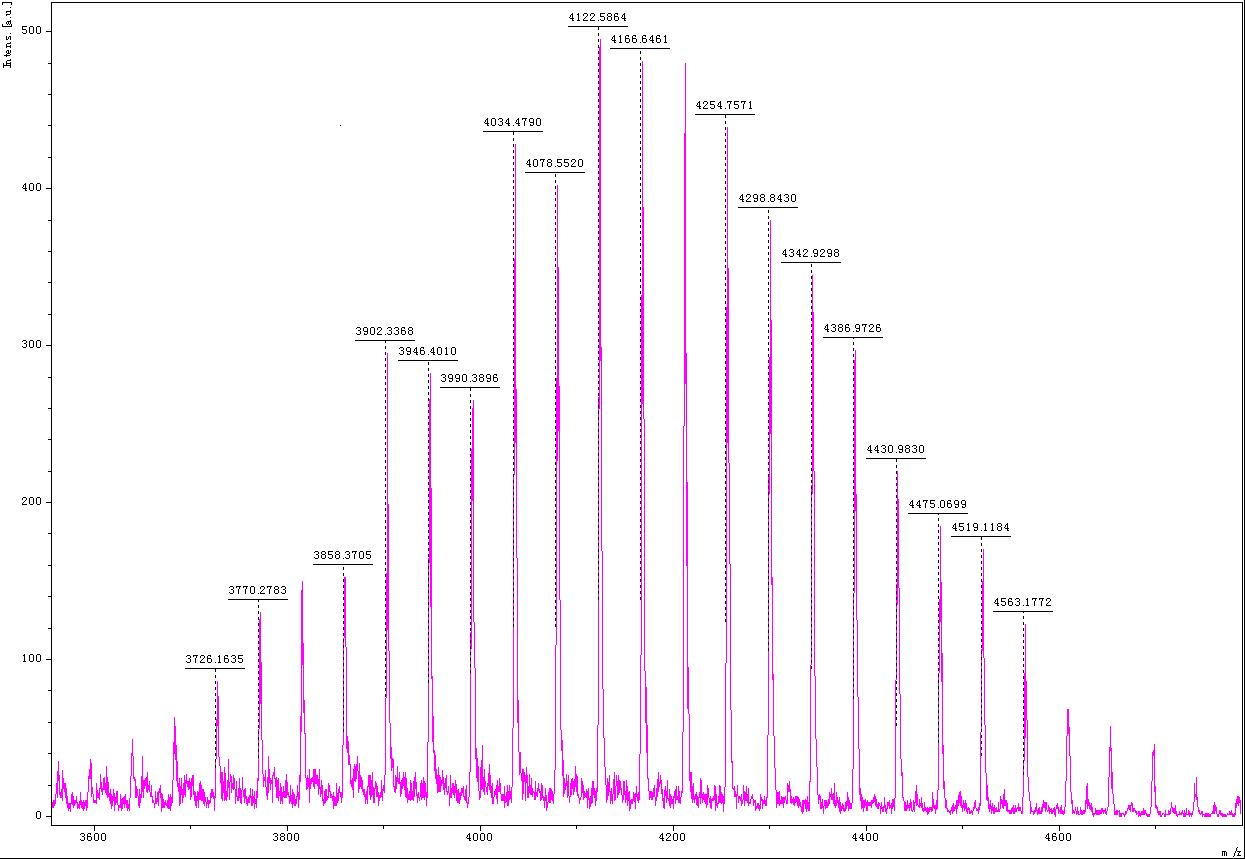


**Supplement figure 2.** Mass spectrum of folate-PEG-CHEMS


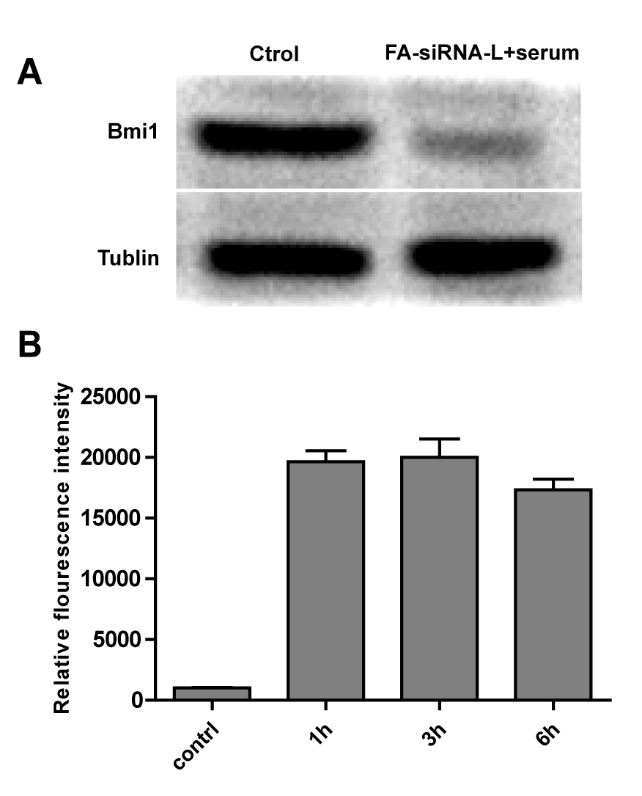


**Supplement figure 3.** (A) Western blotting of KB cells after the treatment of saline FA-siRNA-L in serum. (B) Relative fluorescence intensity detection by FACS.


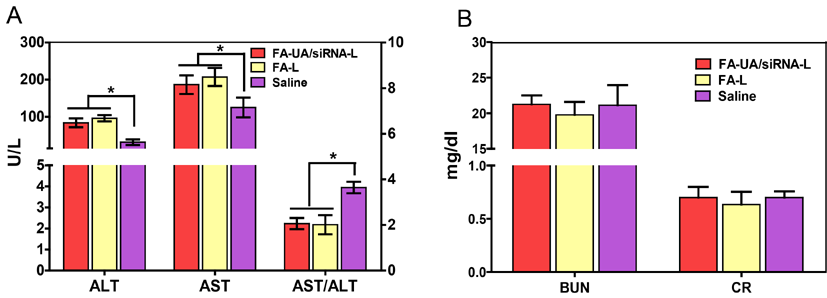


**Supplement figure 4.** Toxicity of cationic liposomes *in vivo.* After Saline, FA-UA/siRNA-L and blank cationic liposomes (320 mg/kg liposome) were injected into Female Kunming mice for 24 h, the plasma was tested with a biochemical analyzer (A). The biochemical criterion of AST, ALT and AST/ALT which respected the toxicity of the liver. (B) The biochemical criterion of BUN and CR which respected the toxicity of the kidney. Data are expressed as mean ± SD (n = 3). *: p<0.05; **: p<0.01.


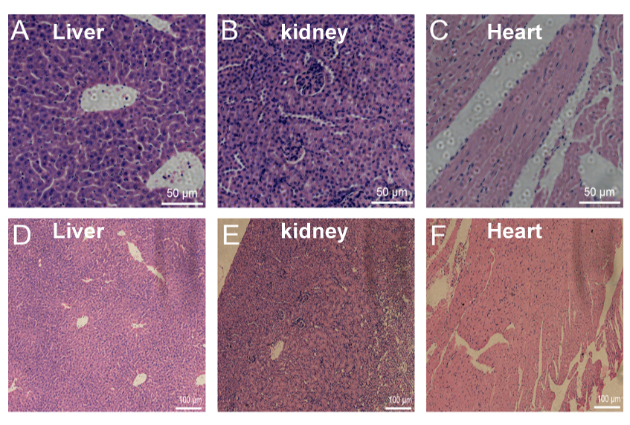


**Supplement figure 5.** H&E staining of liver, kidney, and heart after the treatment of FA-UA/siRNA-L in mice.


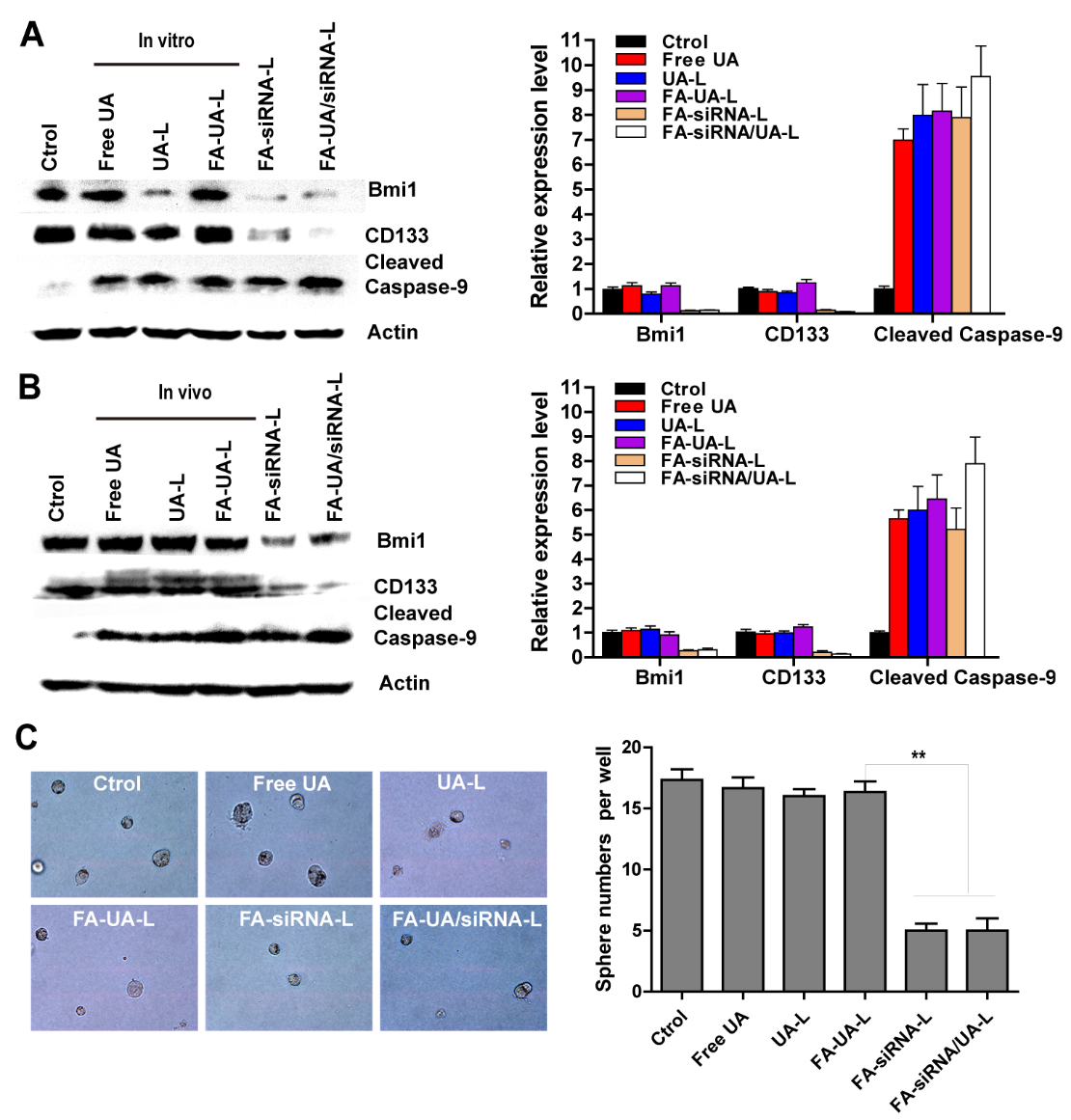


**Supplement figure 6.** Cancer stem cell and apoptosis examination by western blotting of KB cells and mice tumor tissue. (A) Bmi1, CD133, Cleaved caspase-9 protein expression after the treatment of saline, free UA, UA-L, FA-UA-L, FA-siRNA-L and FA-UA/siRNA-L in KB cells. (B) Bmi1, CD133, Cleaved caspase-9 protein expression of tumor tissue after the treatment of different formulations in the xenograft mouse model. (C) Soft agar colony formation assay of KB cells treated with different groups


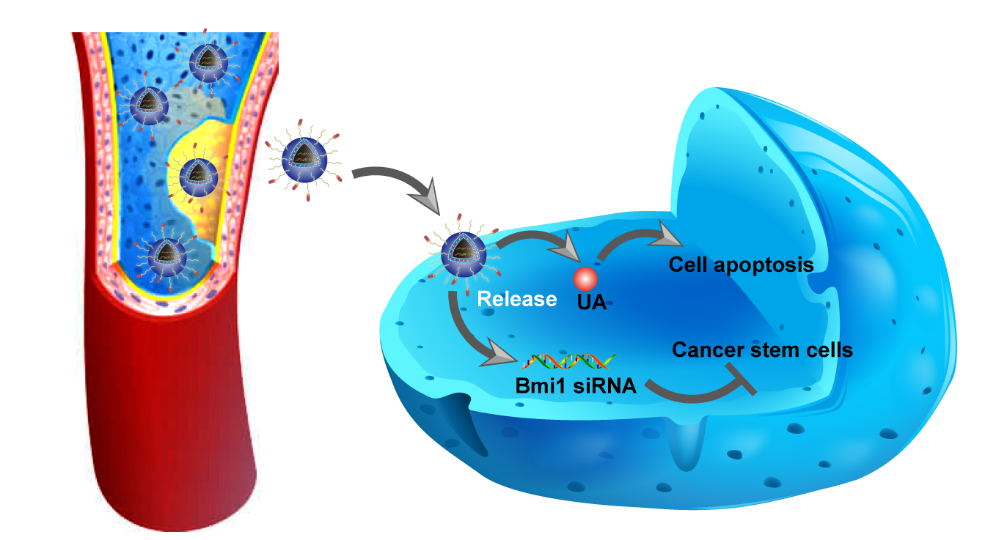


**Supplement figure 7.**The schematic of the anti-tumor mechanism of FA-UA/siRNA-L, UA as an enhancement factor induced tumor cell apoptosis that could make up the ability of apoptosis promotion by Bmi1 siRNA. As a tumor cell self-renewal inhibitor, Bmi1 siRNA could prevent the tumor relapse by inhibiting cancer stem cells.
